# Diffuse Idiopathic Pulmonary Neuroendocrine Cell Hyperplasia: An Uncommon Clinical Challenge

**DOI:** 10.7759/cureus.93666

**Published:** 2025-10-01

**Authors:** Rafaela Faria, Leticia Balanco, Vânia Almeida, Pedro G Ferreira

**Affiliations:** 1 Orthopaedics, Unidade Local de Saúde da Região de Leiria, Leiria, PRT; 2 Pulmonology, Unidade Local de Saúde de Coimbra, Coimbra, PRT; 3 Pathology, Unidade Local de Saúde de Coimbra, Coimbra, PRT; 4 Faculty of Medicine, Universidade de Coimbra, Coimbra, PRT

**Keywords:** diffuse idiopathic pulmonary neuroendocrine cell hyperplasia, dipnech, interstitial lung disease, lanreotide, lung biopsy, somatostatin analogues, vats, video-assisted thoracoscopic surgery

## Abstract

Diffuse idiopathic pulmonary neuroendocrine cell hyperplasia (DIPNECH) is a rare interstitial lung disease characterized by diffuse proliferation of neuroendocrine cells in the airway mucosa and regarded as a preneoplastic condition. It typically affects nonsmoking middle-aged or elderly women and often presents with insidious symptoms like chronic cough, exertional dyspnea, and wheezing. Since these non-specific features may resemble asthma or other obstructive pulmonary airway diseases, the diagnosis is frequently challenging and therefore delayed. Chest high-resolution computed tomography (HRCT) typically shows multiple small pulmonary nodules and a mosaic attenuation pattern, but histological analysis, most reliably obtained by video-assisted thoracoscopic surgery (VATS) biopsy, is the key to establishing the diagnosis.

Herein, we report the case of an elderly woman with a 12-year history of productive cough, exertional dyspnea, and wheezing, initially misdiagnosed as chronic obstructive pulmonary disease (COPD). HRCT revealed multiple small solid nodules and a mosaic attenuation pattern. On physical examination, basal crackles and squeaks were noted on lung auscultation. Following persistent symptoms and inconclusive noninvasive investigations, a VATS lung biopsy was performed, which established the diagnosis of DIPNECH. The patient was treated with systemic corticosteroids and long-acting bronchodilators but developed steroid-related complications without clinical improvement. Follow-up ^68^Ga-DOTA-NOC (gallium-68-labeled 1,4,7,10-tetraazacyclododecane-1,4,7,10-tetraacetic acid-conjugated somatostatin analog) positron emission tomography/computed tomography (PET-CT) showed low-to-moderate somatostatin receptor expression, and therapy with lanreotide was initiated.

This case illustrates the clinical and diagnostic challenges of this uncommon entity and emphasizes the importance of early suspicion, histopathological confirmation, and consideration of somatostatin analogues in selected patients.

## Introduction

Diffuse idiopathic pulmonary neuroendocrine cell hyperplasia (DIPNECH) is a rare interstitial lung disease characterized by diffuse proliferation of neuroendocrine cells in the airway mucosa and is probably underdiagnosed due to the absence of established and validated diagnostic criteria [1]. DIPNECH was first described by Aguayo et al. in 1992 in six nonsmoking patients with cough and dyspnea whose biopsies demonstrated pulmonary neuroendocrine cell hyperplasia [2]. The World Health Organization classifies DIPNECH as a preneoplastic lesion [3]. Pulmonary neuroendocrine cells represent less than 1% of lung epithelial cells and are primarily located in the bronchi, where they are involved in hypoxia detection, immunomodulation, and epithelial regeneration [4].

Airflow obstruction in DIPNECH is thought to result from bronchiolar narrowing and peribronchiolar fibrosis due to the secretion of bioactive substances such as bombesin [4]. Pulmonary neuroendocrine cell hyperplasia may occur as a reactive process - secondary to chronic hypoxia, smoking, or underlying lung disease - or present in an idiopathic form. Idiopathic cases demonstrate higher immunohistochemical expression of p53, Ki-67, and p16 than reactive lesions [5].

DIPNECH occurs predominantly in middle-aged to elderly women, with a mean diagnostic age of 66 years, and is not associated with smoking [6]. Its insidious onset, with chronic cough, wheezing, and exertional dyspnea often leads to misdiagnosis as asthma or chronic obstructive pulmonary disease (COPD), leading to diagnostic delays that may extend beyond a decade. Most patients demonstrate fixed obstructive physiology on pulmonary function tests (PFTs), while high-resolution computed tomography (HRCT) typically shows mosaic attenuation and bilateral pulmonary nodules. Nodules are usually solid, well-defined, and located in middle or lower lung fields; those <5 mm are termed tumorlets, while larger lesions may evolve into carcinoid tumors [5].

Definitive diagnosis requires surgical lung biopsy, with video-assisted thoracoscopic surgery (VATS) regarded as the gold standard. While management is not standardized, inhaled/systemic corticosteroids and bronchodilators are frequently used [3]. Somatostatin analogues (SSA) may provide symptomatic benefit in refractory cases, although adverse effects can limit their use [7]. Long-term surveillance is recommended to detect progression toward neuroendocrine tumors, which occurs in up to 18% of patients [8].

We present the case of an 84-year-old woman with long-standing respiratory symptoms initially misattributed to COPD, who was ultimately diagnosed with DIPNECH after surgical biopsy. Her clinical course highlights the diagnostic delays, therapeutic challenges, and potential role of somatostatin analogues as a treatment option in managing refractory disease.

## Case presentation

An 84-year-old woman, a retired housekeeper, presented with a long-standing history of respiratory complaints. For more than a decade, she had experienced intermittent cough with mucoid sputum, wheezing, and exertional dyspnea corresponding to grade 2 on the Modified Medical Research Council (mMRC) scale. In addition, she reported upper airway symptoms, including chronic rhinorrhea, posterior nasal drip, sneezing, nasal and ocular pruritus, and occasional epiphora. She was initially diagnosed with COPD and treated accordingly, but her symptoms persisted. Her medical history was relevant for type 2 diabetes mellitus, hypertension, dyslipidemia, stage III chronic kidney disease, and chronic non-allergic rhinosinusitis. She had never smoked but reported prior domestic exposure to poultry.

Approximately five years after the onset of symptoms, she was referred to interstitial lung disease clinic for further evaluation. At presentation, she was comfortable on room air with an oxygen saturation of 93%. Chest examination revealed inspiratory Velcro-like crackles at the lung bases and squeaks, while the cardiovascular and systemic examinations were unremarkable.

**Figure 1:**
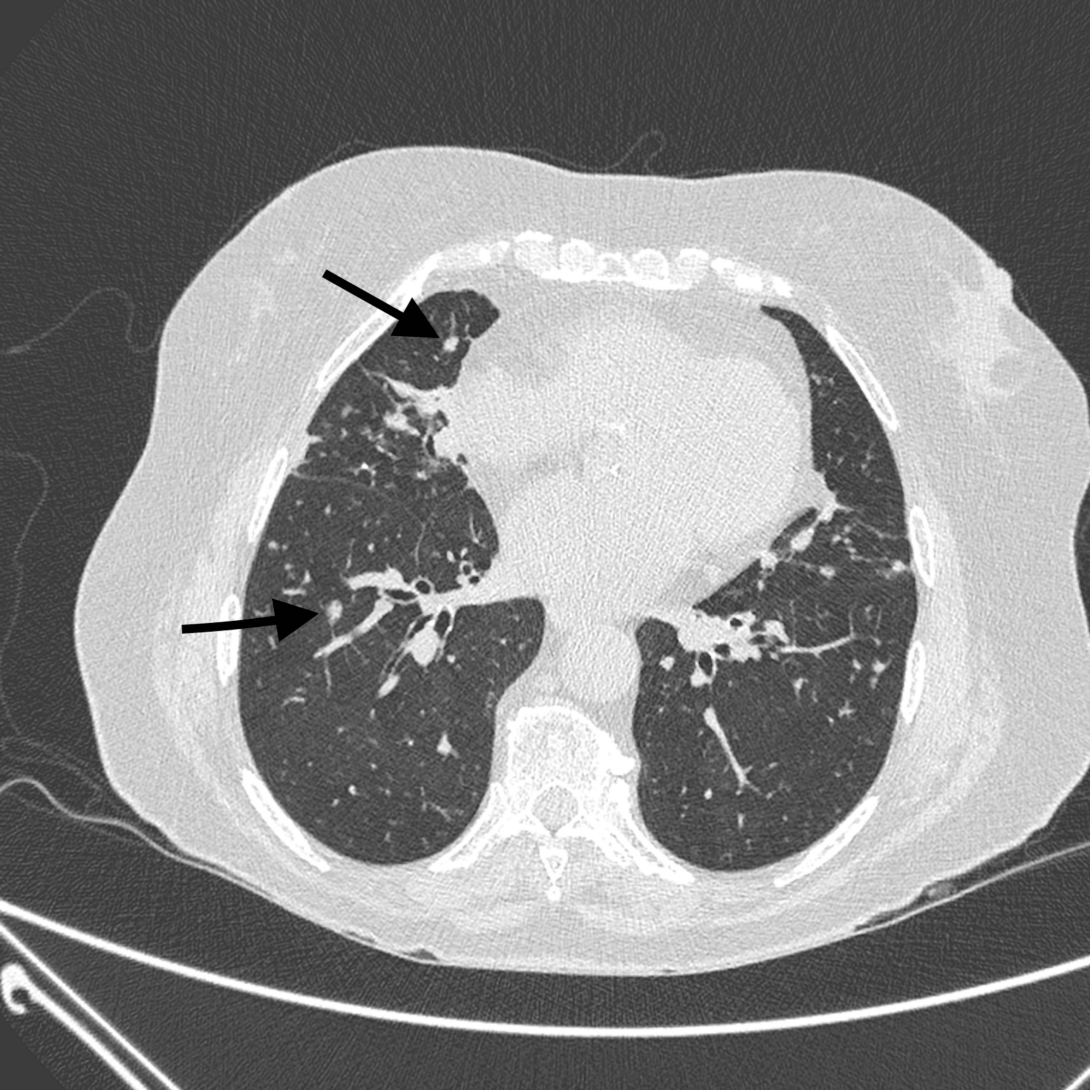
Chest high-resolution computed tomography (HRCT) image at baseline Typical pattern of diffuse idiopathic pulmonary neuroendocrine cell hyperplasia (DIPNECH) with multiple small nodules (black arrows) and areas of mosaic attenuation.

A high-resolution computed tomography (HRCT) of the chest revealed multiple bilateral solid nodules, the largest measuring 6 mm, together with a background of mosaic attenuation (Figure 1). Bronchoalveolar lavage was sterile but showed a lymphocytic predominance (62%), with additional neutrophils (27%), eosinophils (3%), and macrophages (8%). Autoimmune serologies were negative. Pulmonary function tests (PFTs) demonstrated a forced expiratory volume in one second/forced vital capacity (FEV1/FVC) ratio of 0.71, a forced expiratory volume in one second (FEV1) of 65% predicted, a forced vital capacity (FVC) of 71% predicted, and a single breath diffusing capacity of the lung for carbon monoxide (DLCO) of 94% predicted.

**Figure 2:**
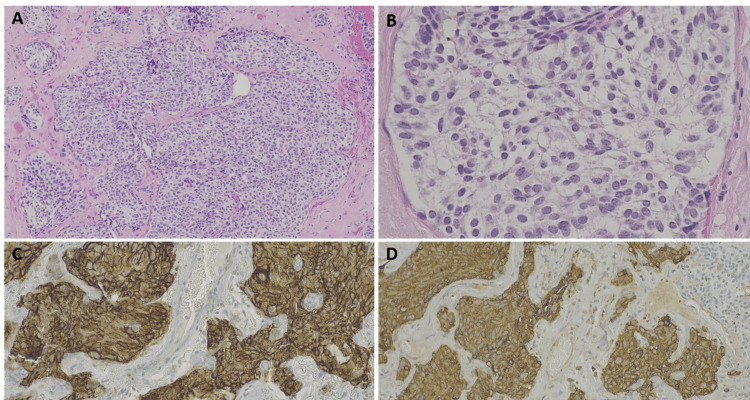
Histopathology of video-assisted thoracoscopic surgery (VATS) lung biopsy showing neuroendocrine cell hyperplasia (A) Nodular proliferation of neuroendocrine cells, original magnification ×40, hematoxylin and eosin (HE). (B) Detail of a nodule showing neuroendocrine cells with round, oval, or spindle-shaped nuclei, salt-and-pepper chromatin, and moderate cytoplasm, ×200, HE. (C) Chromogranin-positive neuroendocrine cells, ×100. (D) CD56-positive neuroendocrine cells, ×100.

Given these findings, a VATS lung biopsy was performed. Histology revealed neuroendocrine cell hyperplasia with a lobulated proliferation of small polygonal cells, positive for chromogranin and Ki-67 (<5%) (Figure 2). These features established the diagnosis of DIPNECH. She was started on systemic corticosteroids (deflazacort 30 mg daily, gradually tapered to 6 mg daily), in combination with inhaled long-acting bronchodilators and corticosteroids.

During the next three years of follow-up, serial HRCT scans remained radiologically stable, but PFTs demonstrated a progressive decline, evolving to a fixed obstructive pattern without bronchodilator reversibility and a gradual reduction in gas transfer capacity (Table 1). Symptomatically, she continued to experience persistent wheezing with minimal relief from inhaled or systemic therapy. Long-term systemic corticosteroid use was complicated by osteoporosis and weight gain.

**Table 1:** Patient’s pulmonary function test results over time FEV1, forced expiratory volume in 1 second; FVC, forced vital capacity; BD, bronchodilator; RV, residual volume; TLC, total lung capacity; DLCO, diffusing capacity of the lung for carbon monoxide.

Parameter		Baseline	Follow-up 1	Follow-up 2	Follow-up 3	Post-treatment
FEV1	Pre-BD (% predicted)	0.65	0.46	0.54	0.55	0.53
	Z score	-1.94	-2.88	-2.39	-2.36	-2.37
	Post-BD (% predicted)	0.64	0.53	0.57	0.57	-
	Z score	-1.97	-2.55	-2.22	-2.26	-
FVC	Pre-BD (% predicted)	0.71	0.64	0.71	0.68	0.66
	Z score	-1.61	-1.98	-1.54	-1.69	-1.73
	Post-BD (% predicted)	0.75	0.71	0.72	0.69	-
	Z score	-1.39	-1.55	-1.49	-1.63	-
FEV1/FVC	Pre-BD (ratio)	0.71	0.55	0.59	0.62	0.61
RV	(% predicted)	-	0.94	0.83	1.1	1.18
TLC	(% predicted)	-	0.85	0.81	0.95	1.01
DLCO	(% predicted)	0.94	0.71	0.73	0.49	0.67

**Figure 3:**
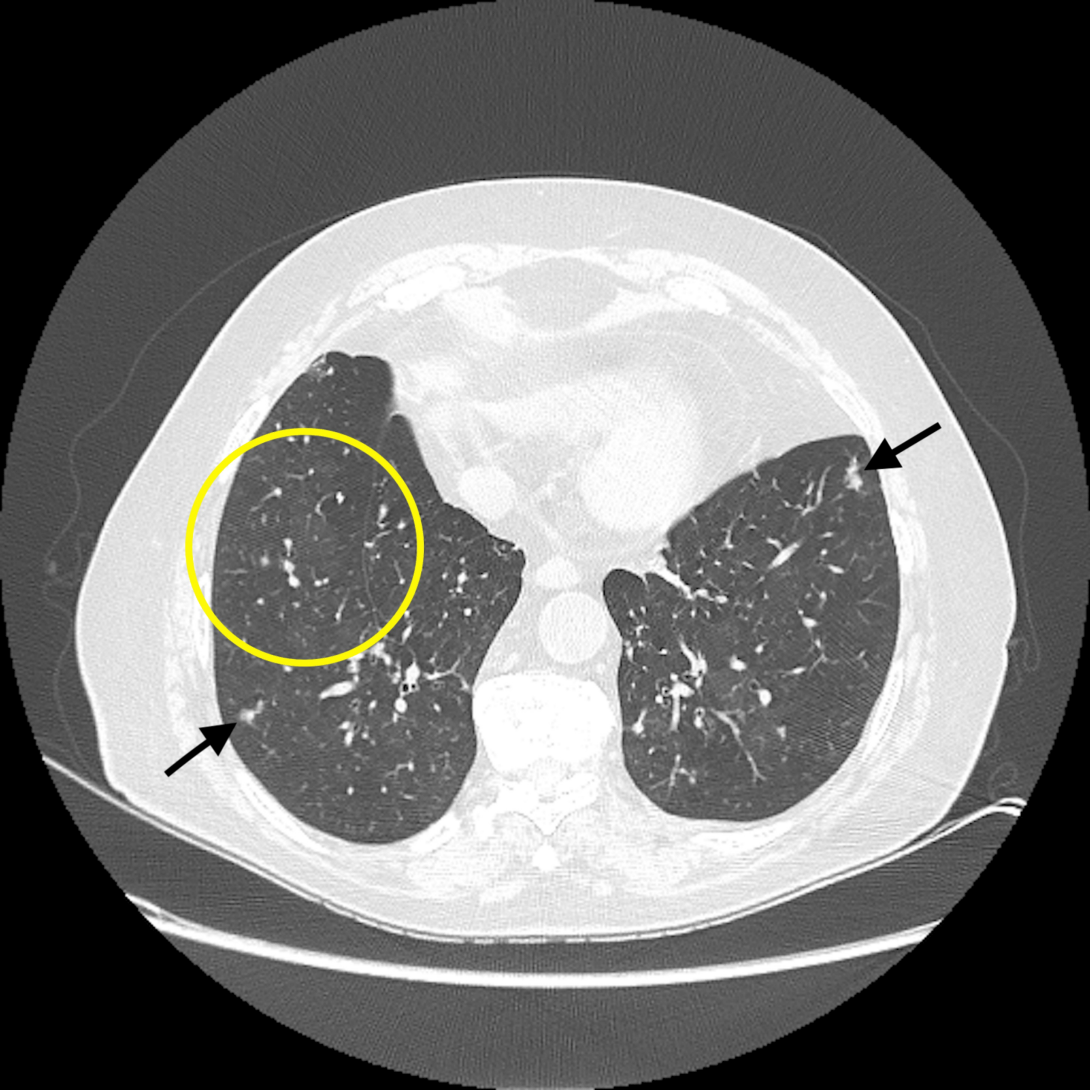
Chest high-resolution computed tomography (HRCT) at reassessment Multiple small nodules (black arrows) and areas of mosaic attenuation (yellow circle).

**Figure 4:**
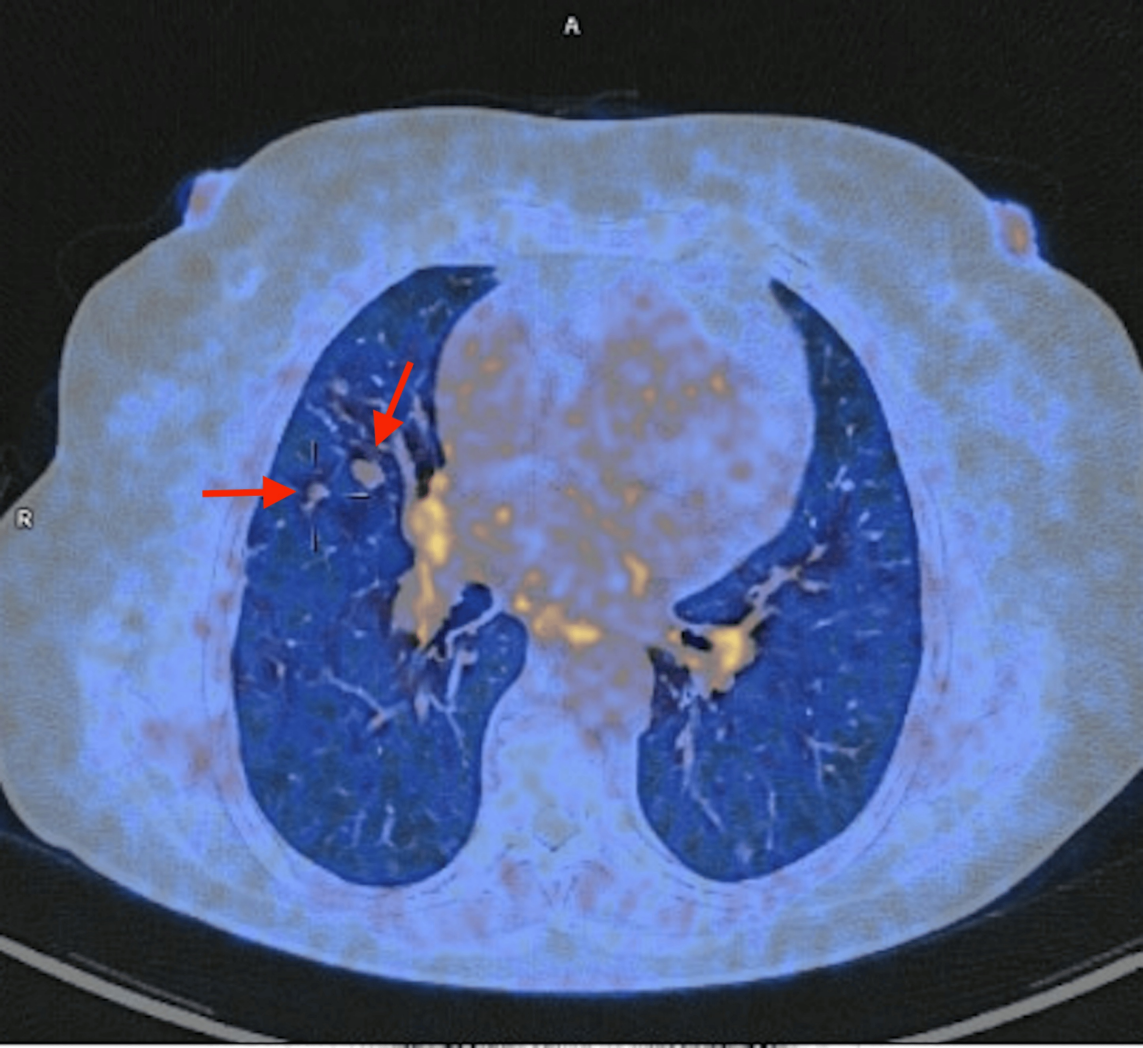
68Ga-DOTA-NOC PET-CT Nodules with low-to-moderate somatostatin receptor expression (red arrows). ^68^Ga DOTA-NOC (gallium-68-labeled 1,4,7,10-tetraazacyclododecane-1,4,7,10-tetraacetic acid-conjugated somatostatin analog); PET: positron emission tomography; CT: computed tomography.

At reassessment, HRCT confirmed the presence of multiple small nodules with unchanged mosaic attenuation (Figure 3). PFTs showed persistent obstruction and further decline in diffusion capacity. Because of refractory symptoms and steroid-related adverse effects, she underwent positron emission tomography/computed tomography (PET-CT) with ^68^Ga-DOTA-NOC (gallium-68-labeled 1,4,7,10-tetraazacyclododecane-1,4,7,10-tetraacetic acid-conjugated somatostatin analog), which demonstrated multiple small nodules with low-to-moderate somatostatin receptor expression (Figure 4).

Considering these findings and emerging evidence supporting the role of somatostatin analogues in this condition, she was started on monthly subcutaneous lanreotide at a dose of 120 mg. After two years of lanreotide therapy, the patient showed sustained improvement in wheezing and cough, a modest enhancement in pulmonary function parameters, and an 18% increase in DLCO.

## Discussion

This case reflects the classical clinical and radiological profile of DIPNECH, differing only in the slightly older age at presentation. Consistent with published reports, the patient was initially misdiagnosed with COPD, a common scenario given the nonspecific symptoms of cough, wheezing, and exertional dyspnea, together with obstructive ventilatory physiology [5,9]. HRCT findings of bilateral pulmonary nodules and mosaic attenuation were central to raising suspicion for DIPNECH in this case, as in prior series [4,9].

Histological confirmation remains essential, and surgical lung biopsy obtained through VATS continues to be regarded as the diagnostic gold standard. In the described case, this approach confirmed idiopathic neuroendocrine cell hyperplasia, allowing a definitive diagnosis. As reported in several cohorts, there was a delay of several years between symptom onset and diagnosis, largely attributable to clinical overlap with asthma and COPD [10,11].

The natural history of DIPNECH is variable. Although progression to overt neuroendocrine tumors is not inevitable, recent studies reported nodule growth in approximately 49% of patients and the development of carcinoid tumors in 18%, with a mean progression time of six years [8]. These findings emphasize the importance of long-term follow-up, with imaging surveillance typically recommended at yearly intervals [12]. Functional assessment is also advisable, although no standardized timing has been established, since respiratory impairment may progress independently of radiological changes.

Therapeutic strategies remain empirical, as available evidence is limited to case reports and small retrospective series. Systemic or inhaled corticosteroids and long-acting bronchodilators are frequently prescribed, but symptomatic control is often unsatisfactory [3]. Somatostatin analogues have emerged as a promising therapeutic option. In a recent series, symptomatic improvement was observed in up to 76% of patients, although adverse events such as gastrointestinal intolerance and hyperglycemia require careful monitoring [7].

In the studied patient, lanreotide was selected due to persistent symptoms despite corticosteroid and bronchodilator therapy, coupled with positive somatostatin receptor expression on ^68^Ga-DOTA-NOC PET-CT. After two years of treatment, the patient demonstrated sustained improvement in cough and wheezing, modest functional gains, and an 18% increase in diffusing capacity, aligning with previously reported benefits of somatostatin analogue therapy [13].

## Conclusions

DIPNECH is an uncommon and often underrecognized condition that may mimic asthma or COPD, particularly in nonsmoking women with persistent respiratory symptoms. Characteristic HRCT findings should prompt further investigation, with histopathology remaining essential for diagnosis. This case reflects the diagnostic challenges of DIPNECH and suggests that somatostatin analogues, such as lanreotide, may offer clinical benefit in refractory disease. Early identification and accurate diagnosis are critical to avoiding unnecessary delays in management. Individualized treatment approaches and systematic follow-up remain key to optimizing outcomes and monitoring for potential progression to neuroendocrine tumors.
